# Absolute and Functional Iron Deficiency in Colon Cancer: A Cohort Study

**DOI:** 10.3390/medicina58091202

**Published:** 2022-09-01

**Authors:** Vlad Făgărășan, David Andraș, Giorgiana Amarinei, Radu Ioan Seicean, Vasile Virgil Bințințan, George Calin Dindelegan, Calin Ioan Căinap

**Affiliations:** 1 1st Surgical Clinic, Department of General Surgery, Iuliu Hațieganu University of Medicine and Pharmacy, 400012 Cluj Napoca, Romania; 2 Department of Medical Oncology, Iuliu Hațieganu University of Medicine and Pharmacy, 400012 Cluj Napoca, Romania

**Keywords:** absolute iron deficiency, functional iron deficiency, colorectal cancer

## Abstract

*Background and Objectives*: Iron is an essential micronutrient for many biological functions and has been found to be intimately linked to cancer biology. Although the effects of increased dietary iron consumption in the development of CRC have been previously investigated in several cohort studies, the available evidence on the involvement of iron deficiency in this process is relatively scarce. Previously published papers did not analyze specific outcomes, such as the presence of biologically aggressive histopathological characteristics, that are associated with the subtypes of iron deficiency. The purpose of this study was to investigate the connection between the development of colorectal cancer and the presence of functional iron deficiency (FID), which is defined as insufficient biological availability of iron in the presence of adequate storage reserves, or absolute iron deficiency (AID), which is defined as severely depleted iron storage levels. *Materials and Methods*: Our paper represents a single center registry-based cohort study. Iron levels were routinely evaluated upon diagnosis of CRC and the collected data were coupled with patient- and tumor-specific data (2018–2022). Spearman’s correlation coefficient and the chi-squared test were used to analyze the association. *Results*: Out of 129 patients, 75 (58.13%) were anemic. AID was identified in 26.35% of cases and FID was encountered in 51.16% of cases. A statistically significant association between FID and lymphatic invasion was encountered. An analysis of the correlation demonstrated a significant association between anemia and right-sided tumor location. *Conclusions*: Functional iron deficiency seems to be independently associated with lymphatic invasion. Although a statistically significant correlation with the T or N stage was not demonstrated, the analysis suggested a potential positive relationship between the presence of FID and more aggressive tumor characteristics.

## 1. Introduction

Colorectal cancer (CRC) is the third most frequently diagnosed and the second most deadly malignant tumor, and the incidence of this disease is expected to increase in the near future [1]. Although CRC is encountered more frequently in highly developed countries, there is a rising incidence in developing nations due to the adoption of “western” dietary habits [2]. In addition, an increased incidence in early-onset CRC has been observed in recent years, possibly due to the widespread introduction of screening programs [3]. Despite recent advances in surgical techniques and the application of increasingly efficient neoadjuvant treatment regimens, CRC continues to be associated with significant mortality rates, especially in advanced stages [4]. The pathogenesis of intestinal malignancies is multifactorial, with both environmental and genetic factors playing a significant role in the development of this disease [5]. Among the multitude of factors implicated in colorectal carcinogenicity, emerging evidence has demonstrated that reduced dietary iron availability and low systemic iron levels may influence colorectal tumorigenesis [6,7]. Although the effects of increased dietary iron consumption in the development of CRC have been previously investigated in several cohort studies, the available evidence on the involvement of iron deficiency in this process is relatively scarce [8,9,10,11]. Iron is an essential micronutrient with a significant influence on various biological functions, several of which are intimately connected to the development of malignant tumors [10,12]. Iron participates in a wide variety of metabolic processes such as erythropoiesis, oxygen transportation, deoxyribonucleic acid (DNA) synthesis, adenosine triphosphate generation, immunological functions, and REDOX cycling. The catalytic form of iron mediates the production of reactive oxygen species via the Fenton reaction and generates oxygen free radicals, resulting in oxidative DNA damage and promoting malignant transformation [12]. Furthermore, iron is necessary for appropriate immunological functions, potentially altering the tumor microenvironment and influencing immune cell-mediated cancer surveillance, both of which may promote tumor development [6]. Therefore, due to the complex interactions of this essential element, a delicate balance of dietary iron must be maintained in order to avoid the negative consequences associated with excessive or deficient intake on the proliferation and development of malignant tumors.

An unexplained diagnosis of iron deficiency anemia (IDA) may be the first indicator for an underlying malignancy in over 8% of cases, especially in the elderly population [13]. Colorectal cancer is frequently associated with the development of IDA, especially right-sided tumors, which have been associated with an unfavorable prognosis. [14] Possible causes of IDA in colorectal cancer include: continuous occult bleeding from the tumor bed, reduced luminal absorption of iron and altered iron metabolism due to the presence of chronic inflammation. Chronic intestinal bleeding results in a severe reduction in or absence of iron reserves, which is defined as absolute iron deficiency (AID) [6]. Chronic inflammation in the presence of malignant tumors leads to a reduction in biologically available iron through an increased production of hepcidin, which causes reduced uptake of iron from the intestine as well as impaired iron release from macrophages, thus resulting in functional iron deficiency (FID) [15].

The purpose of this study was to investigate the correlation between the progression of colorectal cancer and the presence of iron deficiency, either functional or absolute, for a cohort of patients who underwent surgery in a single institution by determining the characteristics of tumors as evidenced by postoperative histopathological analysis.

## 2. Materials and Methods

### 2.1. Patient Selection and Data Collection

Clinical data from 351 patients diagnosed with colon cancer who underwent open or laparoscopic colon resections between January 2018 and April 2022 at the 1st Surgical Clinic, Emergency County Clinical Hospital of Cluj-Napoca were extracted from the hospital records and introduced into an electronic database. All patients with resectable tumors located in the cecum, ascending colon, hepatic flexure, transverse colon, splenic flexure, descending colon, or sigmoid colon were included in the analysis. Patients were excluded from the study if they had received any blood product transfusions or iron administration up to six months prior to diagnosis. In addition, patients that underwent surgery in an emergency setting (tumor perforation or bowel obstruction) were also excluded from the analysis.

Data retrieved from the hospital database included: demographical information; location of the tumor; Union for International Cancer Control, Tumor Node Metastasis (UICC TNM) staging classification according to the postoperative histopathological analysis report; tumor grade; and number of positive lymph nodes. Tumor grade was defined as: low grade or well differentiated (G1), intermediate grade or moderately differentiated (G2), or high grade or poorly differentiated (G3). Patients with tumors located in the cecum, ascending colon, or hepatic flexure of the transverse colon were classified in the right colon category, while patients with tumors located in the splenic flexure, descending colon, and sigmoid colon were classified in the left colon category. Tumors located in the recto-sigmoid junction were considered as superior rectal cancer and were excluded from the analysis. Patients with appendiceal tumors were also excluded from the study.

Hemoglobin, ferritin, transferrin, and serum iron levels were recorded preoperatively 1 to 10 days prior to the surgical intervention. Transferrin saturation (TSAT) was calculated according to the following formula [16]:

TSAT = (Serum iron concentration/Serum transferrin concentration) × 70.9

Anemia was defined according to the World Health Organization criteria as follows: Hb < 13 g/dL (8.1 mmol/L) for adult males and Hb < 12 g/dL (7.4 mmol/L) for non-pregnant adult females [17]. Iron deficiency was classified as AID if plasma ferritin levels were <30 ng/mL and FID if plasma ferritin levels were ≥30 ng/mL and TSAT was <20% [18]. The primary outcome was functional iron deficiency. Secondary outcomes were absolute iron deficiency and anemia.

### 2.2. Statistical Analysis

Statistical analysis was performed using GraphPad Prism version 9.3.0 for Windows, GraphPad Software, San Diego, CA, USA. Descriptive statistics were calculated as means and percentages for continuous variables. Categorical variables were expressed as contingency tables and an analysis was performed using chi-squared and Fisher’s exact tests to compare differences between observed and expected frequencies. Spearman’s correlation coefficient was used to measure the degree of correlation between independent variables. The statistical significance level was set to 0.05 in this study.

## 3. Results

Data were imported from the hospital database on 351 patients who underwent open or laparoscopic colon resections between January 2018 and April 2022. A total of 129 (36.75%) patients met the inclusion criteria and had complete data sets, and thus were included for analysis.

The cohort included 75 (58%) male patients and 54 (42%) female patients. The mean age of all included patients was 69 years old, ranging from 41 to 89 years old. The mean age of patients according to the type of iron deficiency is presented in Table 1.

The mean concentration of hemoglobin was 11.8 mg/dL (CI 95% 11.4–12.2); 58.13% (75) of patients were anemic, while 41.87% (54) of patients had normal levels of hemoglobin. The mean serum iron concentration was 47.4 μg/mL (CI 95% 41.5–53.3). The mean serum ferritin value was 90.1 ng/mL (CI 95% 74.1–106). Absolute iron deficiency was identified in 26.35% (34) of cases and corrected prior to the surgical intervention. Serum transferrin levels had a mean of 255 mg/dl (CI 95% 245–265). The transferrin saturation coefficient was calculated for each patient and had a mean value of 13.5% (CI 95% 11.8–15.2), ranging from 2.82% to 51.8%. Functional iron deficiency was encountered in 51.16% (66) of cases. The tumor-specific pathological characteristics of the included cases are presented in Table 2.

**Figure 1:**
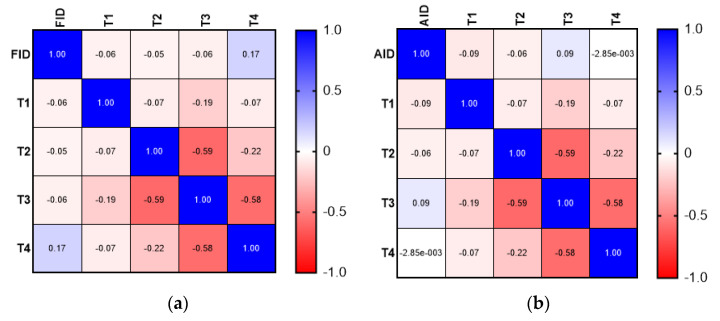
Comparison between FID and AID: (**a**) correlation matrix for FID and T stage; (**b**) correlation matrix for AID and T stage.

**Figure 2:**
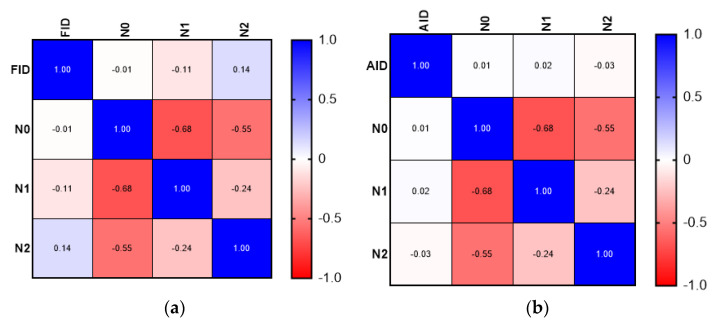
Comparison between FID and AID: (**a**) correlation matrix for FID and N stage; (**b**) correlation matrix for AID and N stage.

**Figure 3:**
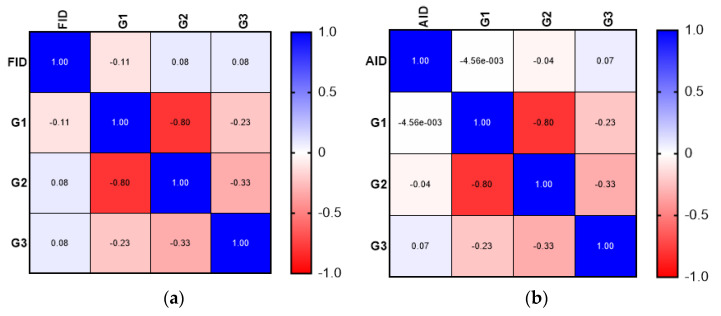
Comparison between FID and AID: (**a**) correlation matrix for FID and tumor grade; (**b**) correlation matrix for AID and tumor grade.

Although the correlation analysis demonstrated a weak positive relationship between the presence of FID and more aggressive tumor characteristics (advanced T stage—T4, N stage—N2), the results were not statistically significant. The correlation analysis did not demonstrate a significant association between FID or AID and tumor grade. The results of the correlation matrix (Spearman’s correlation coefficient) are presented in Figure 1, Figure 2 and Figure 3.

A contingency table analysis using chi-squared and Fisher’s exact tests showed a statistically significant association between FID and lymphatic invasion (OR 2.364; CI 95% 1.019–5.172; *p*-value 0.0451). None of the other investigated parameters demonstrated a statistically significant association with FID.

**Figure 4:**
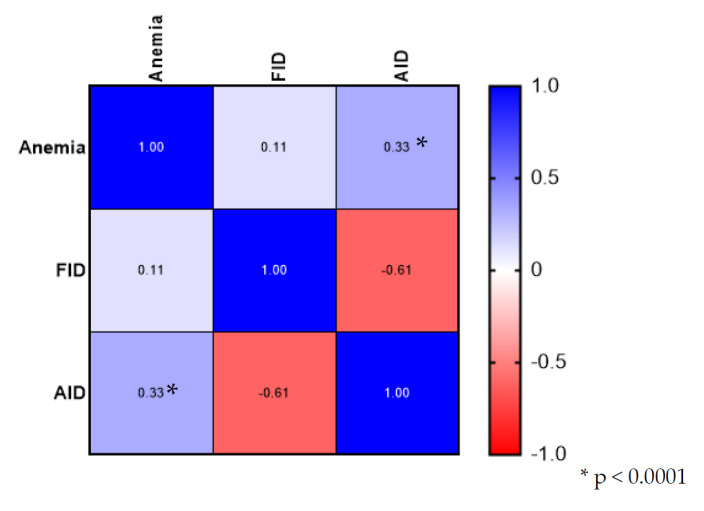
Analysis of correlation between anemia, FID, and AID.

The correlation analysis showed a significant association between anemia and right-sided tumor location (Spearman’s r coefficient 0.37; *p* = 0.00002; CI 95% 0.2026–0.5117). In addition, anemia was associated with absolute iron deficiency (Spearman r coefficient 0.328; *p* = 0.0001; CI 95% 0.1600–0.4784). The correlation matrix for this analysis is presented in Figure 4.

## 4. Discussion

The present study included 66 (51.16%) patients with functional iron deficiency and 34 (26.35%) patients with absolute iron deficiency. While FID is determined by insufficient iron availability for incorporation into erythroid precursors despite the availability of sufficient reserves, AID is determined by severely reduced iron deposits in the bone marrow, liver, and spleen [19]. The principal cause of FID in cancer is the release of pro-inflammatory cytokines such as IL-6, IL-1, TNF-α, and IFN-γ, which amplify hepcidin synthesis and thereby reduce the quantity of iron released into the circulation [7,20,21]. The increased incidence of FID in our cohort could suggest an important inflammatory component that may influence, or be caused by, carcinogenesis and tumor proliferation.

The practical importance of differentiating between AID and FID concerns the way in which iron supplementation is administered in each case [22]. The treatment of AID requires immediate iron administration regardless of the presence of anemia. Conversely, iron supplementation is recommended in the treatment of FID only in symptomatic patients with anemia and should be withheld in patients with elevated ferritin levels [23]. The choice of oral versus intravenous iron therapy is also significant, since oral absorption of iron is reduced in FID through inflammation-related, IL-6-increased hepcidin production in the duodenum, and therefore the effectiveness of this administration method is reduced [24].

Although anemia is a well-known complication of malignant tumors and has been extensively studied, the prevalence of iron deficiency in malignant tumors has been relatively overlooked in the literature [9,10,23,24]. In our study, anemia was identified in 63.2% of patients with FID and 84% of patients with AID. In line with previous studies, a multivariate analysis demonstrated a significant association between tumor location (cecum, ascending colon, and hepatic flexure) and anemia [25,26,27]. Although the sample size for our cohort was relatively small, a statistically significant association was established between anemia and AID. This could be due to occult blood loss, which is more frequent in right-sided colon cancer. Interestingly, FID appeared to have a lesser influence on anemia despite the fact that the number of cases with FID in our cohort was almost double compared to the number of cases with AID.

Previous studies have demonstrated that solid tumors were more frequently associated with both functional and absolute iron deficiency in comparison with hematological malignancies [28,29]. An observational study by Ludwig et al. conducted on 1528 patients with various types of malignant tumors reported an increased incidence of iron deficiency in pancreatic (63.2%), colorectal (51.9%), and pulmonary (50.7%) cancers. Furthermore, the prevalence of ID was significantly associated with cancer stage (*p* = 0.001) and disease status (*p* = 0.001) [23]. Several studies demonstrated that ID with or without anemia was associated with a poorer prognosis, lower disease-free survival, and a reduced response to oncological treatment [7,18,30]. However, to the best of our knowledge, the majority of previously published papers did not analyze specific outcomes associated with AID and FID.

Although our results did not show a statistically significant correlation with either the T stage or N stage, most likely due to the relatively small sample size of the cohort, this finding might be explained by an increased inflammatory response in the presence of a significantly increased tumor burden. However, in our cohort, functional iron deficiency was independently associated with the presence of lymphatic invasion. This association suggests that patients with functional iron deficiency and colon cancer may develop alterations in the immunosurveillance mechanisms and immune cell differentiation processes, which ultimately lead to unfavorable responses to treatment and a poorer prognosis for these patients.

The main strength of the present study was the fact that complete sets of data were available for all cases included in the analysis. The primary limitation of the study was the sample size. Therefore, in assessing the correlations between absolute/functional iron deficiency and tumor-specific pathological factors, the small sample size did not allow us to draw firm conclusions on associations. Despite this fact, our findings suggested that lymphatic invasion seems to be independently associated with functional iron deficiency. Further studies based on a larger number of cases are required in order to further clarify these issues.

## 5. Conclusions

Iron deficiency was highly prevalent in our cohort of patients diagnosed with CRC. Anemia was more frequently encountered in patients with right-sided tumor locations. Functional iron deficiency was significantly associated with higher odds for lymphatic invasion. A large proportion of patients with a normal hemoglobin level were also found to be iron deficient. Further studies on functional and absolute iron deficiency are required in order to more accurately assess possible correlations with colorectal cancer.

## Figures and Tables

**Table 1:** Mean age of patients according to type of iron deficiency.

Type of Deficiency	Mean ± SD (Years)
FID	68.20 ± 9.09
AID	70.15 ± 11.72
Anemia	69.00 ± 10.19
No anemia	70.22 ± 9.34
Overall	69.51 ± 9.82

**Table 2:** Tumor-specific pathological characteristics of the cohort.

Variable	Number of Cases (Percentage)
Location of tumor	
Right colon	45 (34.88%)
Cecum	11 (8.52%)
Ascending	34 (26.35%)
Transverse colon	23 (17.82%)
Left colon	61 (47.28%)
Descending	23 (17.82%)
Sigmoid	38 (29.45%)
Differentiation	
G1	46 (35.6%)
G2	69 (53.5%)
G3	14 (10.9%)
T stage	
T1	3 (2.32%)
T2	24 (18.61%)
T3	79 (61.25%)
T4	23 (17.82%)
N stage	
N0	79 (61.24%)
N1	29 (22.48%)
N2	21 (16.28%)
M stage	
M0	118 (91.48%)
M1	11 (8.52%)
Lymphatic invasion	
L0	90 (69.77%)
L1	39 (30.23%)
Venous invasion	
V0	103 (79.85%)
V1	26 (20.15%)
Perineural invasion	
Pn0	102 (79.07%)
Pn1	27 (20.93%)

## Data Availability

Data supporting the reported results can be obtained via email from the corresponding author.
